# Chemotherapy-induced thrombocytopenia: literature review

**DOI:** 10.1007/s12672-023-00616-3

**Published:** 2023-01-25

**Authors:** Ai Gao, Linlin Zhang, Diansheng Zhong

**Affiliations:** Department of Medical Oncology, Tianjin Medical University General Hospital, No.154, Anshandao, Heping District, Tianjin, 300052 China

## Abstract

Chemotherapy-induced thrombocytopenia (CIT) is a common condition that frequently results in reduced chemotherapy dosages, postponed treatment, bleeding, and unfavorable oncological outcomes. At present, there is no clear suggestions for preventing or treating CIT. Thrombopoietin (TPO) replacement therapy has been invented and used to treat CIT to promote the production of megakaryocytes and stimulate the formation of platelets. However, this treatment is limited to the risk of immunogenicity and cancer progression. Therefore, an unmet need exists for exploring alternatives to TPO to address the clinical issue of CIT. Application of appropriate therapeutic drugs may be due to understanding the potential mechanisms of CIT. Studies have shown that chemotherapy significantly affects various cells in bone marrow (BM) microenvironment, reduces their ability to support normal hematopoiesis, and may lead to BM damage, including CIT in cancer patients. This review focuses on the epidemiology and treatment of cancer patients with CIT. We also introduce some recent progress to understand the cellular and molecular mechanisms of chemotherapy inhibiting normal hematopoiesis and causing thrombocytopenia.

## Introduction

Patients with cancer often suffer from thrombocytopenia. It can be caused by the disease itself or one of its symptoms, but chemotherapy with bone marrow (BM) inhibition is the most common reason. This can lead to fatal bleeding. At present, there are no standardized guidelines for preventing or managing chemotherapy-induced thrombocytopenia (CIT). Therefore, patients with severe CIT usually reduce the chemotherapy dose to reduce the risk of bleeding or the need for platelet transfusion, which may weaken the therapeutic effect and relative dose intensity (RDI) and have a negative impact on the treatment process [1, 2]. Although platelet transfusion can effectively control severe thrombocytopenia for a short time, there are still some problems to be considered, such as allogeneic immunity, infectious pathogen transfer and transfusion reaction [3]. The limitations of platelet transfusions prompt us to look for growth factors that stimulate platelet production and alleviate thrombocytopenia-related bleeding complications, so as to improve the quality of life of patients and reduce or eliminate their reliance on platelet transfusion.

In healthy individuals, mature megakaryocytes developed from multipotent hematopoietic stem progenitor cells (HSPCs) regularly produce functional platelets. The main regulator of megakaryogenesis is known as thrombopoietin (TPO) [4]. In order to promote megakaryocytes and platelet production, TPO replacement therapy has been developed and used to treat CIT, and its effectiveness in treating and preventing CIT has been deeply discussed.

Platelet-producing megakaryocytes are derived from HSPCs in the BM. Hematopoietic stem cells (HSCs) live in BM microenvironment and are regulated by intercellular connections and signaling molecules, which are essential for maintaining a healthy hematological homeostasis [5]. Studies have showed that chemotherapy alters various cells and cytokines in the BM microenvironment significantly, impairing their ability to promote normal hematopoiesis and perhaps causing CIT in cancer patients. Here, we cover the epidemiology and treatment of CIT, and discuss some recent advances in cellular and molecular mechanisms that chemotherapy inhibits normal hematopoiesis and leading to thrombocytopenia.

## Incidence

### Overall incidence of CIT

It is challenging to pinpoint the overall incidence of CIT or for a particular regimen. The incidence of CIT varies greatly between different treatment schemes and the demographic characteristics of patients. Age, type of treatment and type of cancer all affect it in different ways. In early clinical trials, 10 to 68% of patients with solid tumors or hematological cancers experienced CIT [1, 6–8]. A recent retrospective cohort study in the United States (US) of patients with solid tumors or hematological cancers receiving chemotherapy set platelets < 100 × 10^9^/L as clinically significant thrombocytopenia, and estimated the 3-month thrombocytopenia incidence in this study to be 13% for solid tumors versus 28% for hematological cancers [9]. As expected, the incidence of thrombocytopenia in patients with hematologic cancers is higher, and it is worth noting that many of these patients suffered from thrombocytopenia before beginning chemotherapy (Table 1) [9]. In another large-scale observational study conducted in the US from 2010 to 2016, the overall prevalence rate of thrombocytopenia among patients with solid tumors or non-Hodgkin lymphoma(NHL) was 9.7%, which is lower than that of previously published studies [7]. This could be due to the different definitions of CIT used in this research and other analyses. Although platelet counts could not be obtained, this study used diagnostic and program codes to define CIT [7], while the platelet threshold used in other studies is lower than the reference range of a specific laboratory, usually < 100 × 10^9^/L. Patients with low platelet count within one year before starting chemotherapy were also excluded from the evaluation, which was in contrast to other studies, which simply excluded primary thrombocytopenia with non-cancer causes. Some changes in the incidence of CIT, especially among NHL patients, may be explained by this. Therefore, different exclusion criteria and definitions of thrombocytopenia used in these studies lead to differences in CIT incidence.

**Table 1:** The incidence of thrombocytopenia in typical cancer types before chemotherapy [9]

Cancer type	Incidence of thrombocytopenia (platelet count < 100 × 10^9^/L) (%)
All solid tumors	2.8
Lung	3.1
Colon or rectum	1.9
Breast	2.4
Pancreas	3.2
Head or neck	2.4
Ovary	2.4
Prostate	6.1
Bladder	3.7
Uterine	2.7
Melanoma	5.2
All hematologic malignancies	21.2
Non-Hodgkin lymphoma	12.6
Multiple myeloma	27.3
Hodgkin lymphoma	5.8
Acute leukemia	51.8
Other/unknown	30.8

### Incidence of severe CIT

Generally speaking, mild thrombocytopenia has no immediate clinical effects. Once the platelet count of adults is < 5 × 10^9^/L, spontaneous hemorrhage is the leading cause of death [10], which deserves high attention. The National Cancer Institute's Common Terminology Criteria for Adverse Events (CTCAE) is a standard evaluation of possible adverse reactions of hundreds of drugs used in cancer treatment, and it is the most widely used CIT severity grading standard [9]. According to CTCAE (v.5.0), platelet count less than 75 × 10^9^/L is classified as grade 1; between 75 and 50 × 10^9^/L, it is grade 2; if it is between 50 and 25 × 10^9^/L, it is grade 3; under 25 × 10^9^/L, it is grade 4 [11]. Although the bleeding risk and the necessity of platelet transfusion increase with the increase of CIT grade, there are still a few observational studies that have evaluated the relationship between thrombocytopenia and cancer types and chemotherapy regimens according to CTCAE grade. According to early studies, CTCAE grade 3 or 4 CIT occurs in 1% to 56% of patients with solid tumors or hematological malignancies [11]. In the most recent study, 4% and 2% of 15,521 patients with solid tumors developed grade 3 and grade 4 thrombocytopenia, respectively. Of 2537 patients with hematological malignancies, 16% were grade 3 and 12% were grade 4 thrombocytopenia (Table 2) [9]. Although grade 3–4 thrombocytopenia is less common, early identification of low-grade thrombocytopenia may help determine which patients are more likely to have difficulties in the future, and early treatment may be beneficial.

**Table 2:** Incidence of severe CIT (CTCAE grade 3–4) in typical chemotherapy regimens and cancer types [9]

	Incidence of Grade 3 CIT (%)	Incidence of Grade 4 CIT (%)
Chemotherapy regimen		
Gemcitabine	5.5	2.5
Platinum	4.3	2.1
Anthracycline	7.1	3.6
Taxane	2.0	0.8
Other	10.2	7.2
Cancer type		
Lung	5.1	2.6
Colon or rectum	4.3	1.7
Breast	2.7	0.9
Pancreas	4.0	1.4
Head or neck	2.6	0.7
Ovary	4.3	2.1
Prostate	4.5	1.9
Bladder	5.9	3.6
Uterine	4.3	4.0
Melanoma	13.3	5.0
Non-Hodgkin Lymphoma	13.2	9.1
Multiple Myeloma	23.5	18.8
Hodgkin Lymphoma	3.6	2.8
Leukemia and myelodysplastic syndromes	16.3	14.3

### Incidence of CIT in different tumor types and chemotherapy regimens

According to cancer types and chemotherapy regimens, there are significant differences in the incidence and prevalence of CIT. Among chemotherapy regimens based on platinum and gemcitabine, thrombocytopenia bears the greatest burden [1, 6, 9, 12]. The lowest prevalence (8%) was found in patients using taxinoids, followed by anthracyclines (17%) and platinumines (31%), while gemcitabine had the highest prevalence (37%), according to a survey of oncology outpatients in the US [6]. It is worth noting that if receiving multiple chemotherapy drugs, patients in these studies are assigned to a single chemotherapy category according to the order of blood toxicity. For instance, a gemcitabine/carboplatin regimen was only considered as chemotherapy based on gemcitabine rather than platinum. Due to this, the incidence of thrombocytopenia in various chemotherapeutic drug groups was probably underestimated. In terms of tumor types, the incidence of most tumor types (lung cancer, colon cancer, pancreatic cancer, ovarian cancer and bladder cancer) is between 13 and 15%; breast cancer and prostate cancer account for about 10%; melanoma has the highest incidence (21%). Compared to solid tumors, the incidence of thrombocytopenia is higher in multiple myeloma (37%) and NHL (24%) (Table 2) [9], which is consistent with early predictions [6, 7, 12, 13]. In these studies, the incidence of thrombocytopenia is higher in patients with hematological tumors. This is because growth and differentiation of normal HSPCs are blocked [14], so it is significant to highlight that many of these patients suffered from thrombocytopenia before beginning chemotherapy.

CIT is not clearly defined. However, not all post-chemotherapy thrombocytopenias are the same. Clinically, there are two main subtypes of CIT: (1) Nadir CIT. The platelet count of patients reached the lowest point (usually < 50 × 10^9^ cells /L) in the middle of one chemotherapy cycle, and normalized or nearly normalized by the beginning of the next chemotherapy cycle. (2) Persistent CIT, characterized by significant thrombocytopenia, which has not subsided one week or more after stopping chemotherapy at the expiration date [11]. In most cases, CIT is mild and short-lived, and platelet counts will be restored on the first day of the next cycle. However, we must be vigilant, because early identification of low-grade thrombocytopenia may help identify patients who need early intervention, because they are more likely to have complications in the future.

### Management

In patients suffering from severe CIT, the general goal is to prevent bleeding. Vitamin K can be used to correct blood coagulation for patients taking warfarin or those lacking vitamin K-dependent coagulation factors (factors II, VII, IX and X) [15]. Vitamin K can be taken orally, injected subcutaneously or intravenously. In addition, tranexamic acid reduced mortality of bleeding by about one third. In the meta-analysis of using it in elective surgery, blood loss and blood transfusion were reduced by about one third [16]. The side effects of taking this product are minimal, and no research shows that the risk of thrombosis is increased [15]. But in fact, platelet transfusion is the only acute treatment method for severe thrombocytopenia. If the patient is bleeding, or if platelet counts < 10 × 10^9^/L (or < 20 × 10^9^/L in case of fever), platelet transfusion is required to prevent massive bleeding [17, 18]. However, transfusion-related acute lung injury is still an obstacle to effective blood transfusion for multiparous women [19]. Although single-donor platelets are often considered superior to platelets obtained from multiple donors, a randomized trial has shown that they are no more effective in reducing allogeneic immunity or transfusion intolerance [20]. Therefore, it is of great significance to apply hematopoietic growth factor to promote platelet recovery.

Oprelvekin (IL-11) was approve by the FDA to treat CIT [21]. A series of clinical trials have shown that recombinant human IL-11 (rhIL-11) is widely used in the treatment of III or IV thrombocytopenia. Although the use of rhIL-11 does increase the number of platelets and reduce the risk of bleeding, however, its side effects can not be ignored. A clinical trial has shown that the use of rhIL-11 can directly or indirectly induce cardiotoxicity, and the use of rhIL-11 in older and frail patients may cause or aggravate existing heart failure diseases (such as potential heart disease, pulmonary infection, etc.) [22] Thus it can be seen that the related toxicity caused by IL-11 often exceeds its limited efficacy.

The development and differentiation of megakaryocytes are affected by many cytokines. TPO is the main cytokine which regulates the development and maturation of megakaryocytes [4]. Recombinant TPO has been produced and is used to treat many thrombocytopenic diseases to promote the production of megakaryocyte and increase the formation of platelet. Early clinical trials were encouraging [23, 24], but research was discontinued due to cross-reactivity of anti-TPO autoantibodies with endogenous TPO resulting in drug-induced thrombocytopenia [25]. Therefore, the second generation of TPO molecules-TPO receptor agonists (TPO-RAs) without immunogenicity-such as romiplostim, eltrombopag and avatrombopag, were developed one after another. They are approved for the treatment of chronic immune thrombocytopenia (ITP) and severe aplastic anemia (SAA) [26–29]. There is currently no US FDA approval for any of these medications to treat CIT, however romiplostim, eltrombopag and avatrombopag have all been the subject of numerous studies testing their efficacy in the management of CIT (Table 3).

**Table 3:** Comparison of the various TPO-RAs [11]

	Romiplostim	Eltrombopag	Avatrombopag
Molecular structure	Fusion protein	Small molecule	Small molecule
Site of binding	Extracellular domain of c-mpl	Transmembrane domain of c-mpl	Transmembrane domain of c-mpl
Route of administration	Subcutaneous	Oral	Oral
Dosing frequency	Weekly	Daily	Daily or less frequently than daily
Main function	CIT treatment (Increased platelet counts, reduced chemotherapy dosage reduction rate, treatment delay, bleeding and platelet transfusion)	CIT prevention (Shortened the platelet recovery time and reduced dose delay/reduction)	CIT prevention (Increased the nadir platelet count)
Non-benefit group	Patients with bone metastases, patients who have received pelvic radiotherapy before	Patients who had nadir CIT only in the middle of a chemotherapeutic cycle	Not clear yet

#### Romiplostim

Romiplostim is a fusion protein, which consists of the Fc region of human IgG1 antibody and a sequence of 14 amino acids, and binds to the extracellular domain of the thrombopoietin receptor c-mpl [11]. Early case reports [30, 31] and observational studies [32, 33] show that romiplostim can safely and effectively increase platelet count of CIT patients induced by different tumor types and different chemotherapy regimens, and maintain proper platelet counts in some patients for many years. In the latest phase II randomized trial, compared with untreated CIT (platelet counts < 100 × 10^9^/L for more than 4 weeks despite delayed chemotherapy or dose reduction), platelet counts normalized in 60 patients with solid tumors receiving romiplostim with a success rate of 85% (44/52), which allowed them to continue chemotherapy and maintain romiplostim [34]. However, these studies on the treatment of CIT by romiplostim were limited to case types and small single-center research, until a large-scale observational cohort study involving 173 CIT patients with solid tumors, lymphoma or myeloma [35]. Among the patients with solid tumors who received a range of different chemotherapy treatments, they found that romiplostim was successful in managing CIT, with the support of romiplostim, with 98% (170 of 173 patients) were able to continue their chemotherapy. Romiplostim treatment increases the median platelet count in the cohort more than twice (from 54 × 10^9^/L to 112 × 10^9^/L), enabling 79% of patients with solid tumors to continue treatments without having to reduce or delay the chemothrapy dose due to thrombocytopenia, and 89% of patients could complete treatment without platelet transfusions [35]. Additionally, they also proved that CIT was resistant to romiplostim therapy in three cases: (1) bone marrow invasion, (2) prior pelvic irradiation, and (3) prior temozolomide [35]. A phase II clinical trial found that romiplostim was completely effective for patients with liver metastasis of cancer [32]. These studies have concluded that romiplostim is effective in CIT treatment of solid tumor patients, which is characterized by increased platelet counts, low reduction rates of chemotherapy dose, delayed treatment, bleeding and platelet transfusion. Thrombocytopenic cancer patients who may benefit from romiplostim include patients without bone metastases, patients who have not received pelvic radiotherapy before, and patients whose liver is involved in cancer.

#### Eltrombopag

Eltrombopag is small molecule that bind to the transmembrane part of the receptor [11]. The main trials of eltrombopag in CIT evaluated its application in the prevention of CIT, compared with the published data for romiplostim, which mainly examines its application in CIT treatment. This can be traced back to a phase 2 study in 2010 [36]. The primary endpoint of this study was the difference in platelet count from day 1 to the lowest platelet point in cycle 2. From day 2 to day 11 of the 21-day chemotherapy cycle with the carboplatin and paclitaxel regimens, these 183 patients were randomly given placebo or eltrombopag 50 mg, 75 mg or 100 mg/day for two or more cycles. 134 patients completed at least two cycles. Patients treated with eltrombopag did have higher platelet counts at the start of subsequent treatment cycles, but it did not reach the primary end point [36]. This requires additional studies to explore the optimal dose and duration of eltrombopag in patients receiving BM suppression chemotherapy. In another phase 1 clinical trial, 26 pancreatic cancer patients who received gemcitabine monotherapy, gemcitabine combined with cisplatin or gemcitabine combined with carboplatin were randomly assigned to receive eltrombopag 100 mg or placebo every day for 5 days before and after chemotherapy at a ratio of 3:1. The mean platelet nadirs of patients treated with eltrombopag were considerably higher, with 14% of patients experiencing chemotherapy dose reductions or treatment delays that were significantly lower than 50% of controls [37]. The results of this study need to be further verified in phase II clinical trials. The recent study involved 75 solid tumor patients who were randomized 2:1 treated with eltrombopag 100 mg or placebo while receiving gemcitabine plus cisplatin, carboplatin or gemcitabine monotherapy. Only 26 of the recruited patients finished all of the chemotherapy treatment cycles required for the trial. Although the incidence of grade 3 or 4 CIT in both groups were generally high, patients receiving eltrombopag have higher platelet counts, lower incidence of grade 3 or 4 CIT, faster recovery of platelet count, and less dosage reduction/treatment delay or missing doses due to thrombocytopenia [38]. Overall, the treatment of eltrombopag shortened the platelet recovery time and reduced dose delay/reduction caused by thrombocytopenia. However, eltrombopag administration was unsuccessful in patients who had nadir CIT only in the middle of a chemotherapeutic cycle.

#### Avatrombopag

Avatrombopag, like eltrombopag, is an oral TPO receptor agonist, which promotes thrombopoiesis of HSCs, megakaryocyte precursors, and megakaryocytes [39]. Avatrombopag has completed a phase 3 randomized controlled trial in the treatment of CIT in 122 patients with non-hematological malignancies [40]. For 5 days prior to and after chemotherapy, the patient received either avatrombopag 60 mg daily or placebo in a 2:1 ratio [40]. According to this study [40], avatrombopag did not reach the primary endpoint of prevention of platelet transfusion, chemotherapy dose modification or treatment delay. Only 69.5% of patients receiving avatrombopag achieved the primary endpoint, compared to 72.5% of patients receiving placebo. However, avatrombopag did increase the nadir platelet count (51 × 10^9^ cells/L in avatrombopag group vs 29.1 × 10^9^ cells/L in placebo group). Patients with a previous CIT history and patients who had previously received more than two chemotherapy regimens were excluded from this trial, which had some limitations. It is necessary to conduct avatrombopag evaluation on the more durable CIT population.

#### Trilaciclib

Trilaciclib is an intravenous CDK 4/6 inhibitor given before chemotherapy to protect HSPCs from chemotherapy-induced damage [41, 42]. Based on the results of previous clinical trials, Trilaciclib has been approved for the treatment of BM suppression caused by chemotherapy for extensive small cell lung cancer [43–45]. Grade 3 or 4 hematologic adverse events were reduced by half in trilaciclib-treated patients when compared to placebo, with grade 3 or 4 thrombocytopenia occuring in 18% of trilaciclib-treated patients versus 33% of placebo-treated patients before chemotherapy [46]. Based on the existing data and research results of colorectal cancer, breast cancer and bladder cancer, this type of drug can be considered as a feasible treatment strategy through non-selective prevention of chemotherapy-induced BM suppression. However, in these studies the number of patients was small, and further investigation is warranted.

Blood transfusions and growth factors can help lessen anaemia and neutropenia. But erythropoiesis stimulating agents can not be used to treat anaemia due to concerns that they may hasten tumor progression and deaths in certain types of solid tumors [47]. In contrast to erythropoietin receptor, the expression level of TPO receptor in cancer cells is very low or undetectable [48]. Hematological malignancies, however, are an exception to this. An elegant study by Rauch et al. proposed the MPL^hi^ state as a marker for more severe thrombocytopenia at diagnosis and linked thrombopoietin scavenging by MPL^hi^ leukemic blasts to thrombocytopenia in AML patients [49]. In a number of trials involving MDS patients with thrombocytopenia, treatment with romiplostim and eltrombopag resulted in increased levels of leukemia cells in the blood compared to placebo [50]. And, because of the danger of progression to leukemia, only a few trials for this indication are currently underway [51]. Therefore, an unmet need exists for exploring for alternatives TPO to address the clinical issue of CIT. Understanding the underlying mechanisms of CIT is helpful for the application of appropriate therapeutic drugs.

## Mechanism

### Response of HSPCs to chemotherapy

#### Differentiation of megakaryocytes and platelets

Megakaryocyte is the precursor of platelet, and it originates from HSPC in BM. HSCs are defined as the most primitive cell populations with self-renewal and differentiation potential [52]. They can differentiate and give rise to diverse blood cell types. According to the conventional theories, there is a distinct hierarchy between HSC and its progeny cells and mature blood cells. The hematopoietic differentiation model is based on the identification of various cell populations of the hematopoietic system. HSCs and multipotent progenitors (MPPs) are differentiated downward into common myeloid progenitors (CMPs) [53]. Following HSCs and MPPs, CMPs and common lymphoid progenitors [54] established themselves as the initial nodes of lineage differentiation. By differentiating into megakaryocytic-erythroid progenitors, MEPs and granulocyte-monocyte progenitors eventually differentiate into mature megakaryocytes, red blood cells and other myeloid cells. Chemotherapy causes cytopenia, which has clinical effects including thrombocytopenia, since it impairs normal hematopoietic function.

#### Chemotherapy induces acute BM injury by causing HPC apoptosis

According to some studies, acute BM injury occurs soon after chemotherapy due to inducing apoptosis of hematopoietic cell [55, 56]. Because most of HSCs are quiescent and better at repairing DNA damage, they are more resistant to chemotherapy-induced apoptosis than proliferating HPCs [57, 58]. Pawel et al. reported that injection of cytotoxic drug 5-FU into mice resulted in massive apoptosis of BMCs and in vitro clonogenic assays revealed that it had an impact on the regeneration of megakaryocytic precursors [56]. A study in 2003 showed that incubation of busulfan with BM mononuclear cells failed to induce HSCs apoptosis although it significantly inhibited hematopoietic function [59]. In addition, cisplatin destroys normal hematopoiesis by reducing the production of colony-forming unit granulocytes/macrophages (CFU-GM) [60, 61]. Another study showed that increasing DNA fragmentation in BM cells from rat models receiving carboplatin treatment was linked to rising HPCs apoptosis [62]. Apoptosis of HPCs and myelosuppression are also linked to increased DNA fragmentation, according to two further investigations using carboplatin [63, 64]. Therefore, the acute BM injury is mainly due to the apoptosis induced by chemotherapy in the rapidly proliferating HPCs [65]. In this case, the hematopoietic system’s homeostasis is restored by HSCs going through self-renewing and differentiation to replenish HPCs, and then produce mature blood cells, including platelets. Since megakaryocyte differentiation and maturation can be stimulated by TPO, which is frequently used in clinical practice to encourage the recovery of BM hematopoietic function in patients after cancer therapy. Thus, most cancer patients who receive chemotherapy with or without TPO can quickly recover from acute BM suppression. According to the studies presented above, chemotherapy may induce apoptosis of progenitor cells leading to reduced differentiation to megakaryocytes and platelets, while upstream HSCs can supplement progenitors by self-renewal and differentiation into downstream progenitors. This may explain why some patients have brief, mild CIT and their platelet counts recover in the next cycle of chemotherapy.

#### Chemotherapy induces long-term (LT)-BM injury by reducing the self-renewal and proliferation ability of HSCs

But in certain cases, thrombocytopenia persists and does not recover even in the subsequent chemotherapy cycle, and these patients experience LT-BM injury after chemotherapy due to HSC damage [66]. LT-BM injury is more likely to occur when carboplatin, busulfan and bis-chloronitrosourea are treated [59, 67]. In the case that chemotherapy does not affect the self-renewal ability of HSCs, they can undergo self-renewing to replace the depleted HSCs, so induction of HPCs apoptosis may have little effect on LT-BM damage even though it may lead to acute BM injury induced by chemotherapy [68]. Chemotherapy mainly inhibits the replication and self-renewal of HSC by inducing the senescence of HSC, which leads to the decrease of HSC reserve and eventually leading to LT-BM injury. HSC self-renewal has been shown to be impaired in patients and animals following treatment with various chemotherapeutic agents. For instance, after receiving chemotherapy, mice BM HSCs produced fewer colony-forming units-spleen (CFU-S) and regenerative units after transplantation into the recipient BM [69–71]. In the same way, the patients who received autologous transplantation after dose-intensive chemotherapy showed similar abnormalities in self-renewal ability and long-term reproduction ability of HSC. Though the precise mechanism is not yet clear. HSCs are relatively more sensitive to oxidative stress, which may be partly because they are usually located in a hypoxic environment in the HSC niche and maintain in a quiescent state. As a result, a mild increase in ROS has the potential to reduce the self-renewal capacity of HSCs by triggering HSC senescence, which can result in premature exhaustion of HSCs and LT-BM injury, which can result in permanent thrombocytopenia [72–74]. It has been suggested that LT-BM damage brought on by chemotherapy contributes to the pathophysiology of BM suppression by inducing HSC senescence as a result of increased ROS generation. Bikul Das et al. found that cisplatin can hinder normal hematopoiesis by generating oxidative stress in BM in an in vivo mouse model [60]. These studies demonstrated that chemotherapy inhibited hematopoiesis by promoting oxidative stress and reducing the self-renewal capacity of HSC. As is well known, cytokines are factors that can induce the development and differentiation of HSCs. Transforming growth factor β1 (TGF-β1) plays an important role in maintaining the quiescent state of HSCs [75, 76]. The activation of TGF-β1 will inhibit the proliferation of HSCs, which may be related to serious complications such as pancytopenia [77–79]. It has been demonstrated that etoposide exposure of human BM stroma cells causes ROS/and matrix metalloproteinase-2(MMP-2) dependent activation of TGF-β1, which can impact hematopoiesis [80]. This suggests that the TGF-β1 intracellular signal transducer may indirectly contribute to CIT. Above all, the loss of self-renewal ability and decreased proliferation of HSCs and exhaustion of HPCs account for thrombocytopenia in cancer patients.

Most of these studies only focused on the effect of chemotherapy on HSPCs, and did not thoroughly examine the differentiation of megakaryocyte-platelet lineage, so they were unable to clearly address the causes of CIT. In addition, HSC is a heterogeneous cell population. Numerous studies have showed that the HSC cell population contains a subpopulation of HSC (megakaryocyte-biased HSCs) that is biased towards megakaryocyte differentiation, bypassing the conventional intermediate progenitor stage and directly differentiating into megakaryocytes [81–84]. And it was verified that when this subset of HSCs is activated in response to inflammatory stress or acute thrombocytopenia, platelet count is successfully restored [82, 85], this suggests that this subset of HSC can be used for short-term platelet reconstruction, offering an important therapeutic target for platelet reconstruction following chemotherapy. Therefore, when researching CIT in the future, we need to pay closer attention to the changes in the upstream HSC population.

### Chemotherapy-induced bone marrow niche alterations

In adults, HSC resides in a highly organized BM structure, which is called niche. It is partly produced by endothelial cells and stromal cells, which determines their behaviour [86]. Any disruption to BM niche would affect the quantity and capacity of HSC. Chemotherapy not only consumes HSC, but also damage its microenvironment by destroying endothelial cells and stromal cells [87–89]. Studies have shown that chemotherapy agents can induce apoptosis of osteoblasts and reduce differentiation of osteoblasts [90–92]. Studies have also shown that doxorubicin and etoposide can increase the directional differentiation of BM mesenchymal stem cells into adipogenic lineage, thus reducing bone mass [93]. After chemotherapy, most niche cells are reduced while adipocytes increased [94], which is directly linked to decreased bone mass. Most HSCs in the BM are located near the sinus blood vessels, which are in direct contact with HSCs, and their consumption leads to the loss of HSCs [95]. It was known for a long time that chemotherapy causes BM endothelial cells' physical and functional integrity to be compromised [87, 88]. Previous studies found that megakaryocytes are localized in vivo to sinus-shaped BMECs, where they form unique transendothelial pseudofeet, or migrate through BMECs, where they release platelets directly to the bone marrow-intravascular sinus cavity or lung capillaries [96, 97]. Megakaryocyte interaction with junctional BMEC adhesion molecules may be necessary for thrombopoiesis. Scott et al. reported that mice treated with 5-FU result in the reduction of polyploid megakaryocytes, accompanied by the corresponding reduction of intact sinusoidal vessels, indicating that the destruction of the vascular niche damaged the maturation and polyploidization of megakaryocytes [98], causing CIT. In addition, SDF-1 and FGF-4, which enhances the interaction between megakaryocyte and bone marrow vascular niche, diminished thrombocytopenia after myelosuppression induced by chemotherapy [98]. Therefore, progenitor-active chemokines can avoid life-threatening thrombocytopenia after chemotherapy by rebuilding hematopoietic system, and provide a new treatment strategy for CIT. In addition, multiple solid tumors prefer BM as a metastatic site, and it is associated with cytopenia [99]. A recent study using mouse mammary tumour cells showed that metastases grow and rapidly reshape local blood vessels through widespread germination, thereby establishing a tumor-supported microenvironment [100]. Therefore, in the future, it will be important to examine how tumor cells affect the BM microenvironment while studying how chemotherapy affects it.

### Drug-induced immune thrombocytopenia (DITP)

Cytotoxic chemotherapy often inhibits normal hematopoiesis. On the other hand, CIT is characterized by increased platelet clearance by mononuclear phagocytes, it is usually mediated by immune mechanism involving drug-dependent antibodies, which may also induce direct platelet destruction [101, 102]. This condition is known as drug-induced immune thrombocytopenia (DITP), which is not uncommon, but it can be difficult to diagnose. Many chemotherapy regimens can cause DITP, but oxaliplatin is the most common one. Oxaliplatin is the third-generation platinum analogue, which is commonly used in gastrointestinal malignant tumors with 5-fluorouracil (5-FU) based regimens [103]. Sudden onset of isolated severe thrombocytopenia is the main feature of oxaliplatin-induced immune thrombocytopenia (OIIT) [104]. The mechanism of DITP is still unclear. The current hypothesis is that antibodies against specific platelet glycoproteins, such as glycoprotein IIb/IIIa (GPIIb/IIIa) complex proteins are considered to play a role in DIIT [105]. It is reported that GPIb/IX or GPIa/IIa are also potential targets [105]. Hapten-associated antibody response, autoantibody and/or immune complex formation are among suspected mechanisms for OIIT [106]. Early realization of DITP might save lives through easier interventions like platelet transfusion for symptomatic severe thrombocytopenia or drug withdrawal. In addition, repeated infusion of oxaliplatin might lead to immune reaction in sensitized patients. The patients with DIIT were reported to have high cumulative oxaliplatin doses [107]. We should also be alert to thrombocytopenia after long-term exposure, because the median exposure time of oxaliplatin was reported to be 10 cycles [104].

## Conclusion

**Fig. 1:**
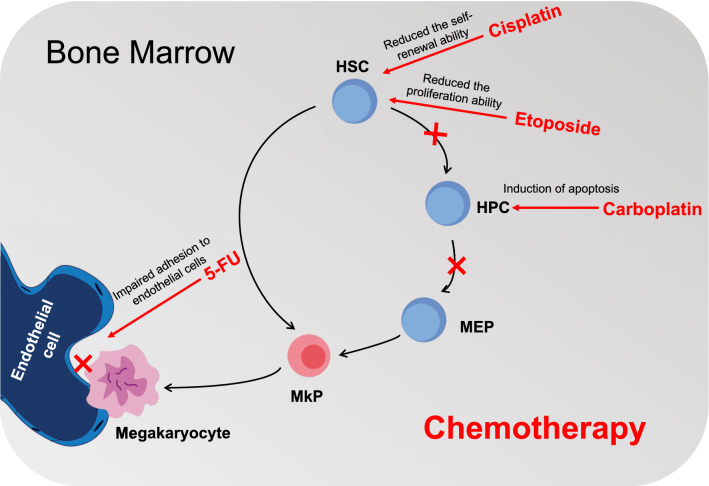
Refined model of native megakaryocytic differentiation and role of chemotherapy in BM. HSCs can differentiate and give rise to diverse blood cell types. HSCs are differentiated downward into HPCs. Following HSCs and HPCs, MEPs eventually differentiate into mature megakaryocytes. Chemotherapy agents cause thrombocytopenia, since it impairs normal hematopoietic function on the different stages of cell differentiation. *HSC* hematopoietic stem cell; *HPC* hematopoietic progenitor cell; *MEP* megakaryocytic-erythroid progenitor; *MkP* megakaryocytic progenitor

CIT is a common complication of cancer treatment, which may endanger the results of oncology. Several studies describing the safety and effectiveness of using TPO-RAs to manage CIT have just been published, despite the fact that there are currently no U.S.FDA-approved agents available to treat CIT. To address the clinical issue of CIT, there is an unmet need for alternatives TPO research due to the risk of progression to leukemia in MDS patients. Understanding the underlying mechanisms of CIT may help to apply appropriate therapeutic drugs. Our previous research discovered an excessive production of IL-4 by BM endothelial cells and found that this had a striking role in suppressing megakaryocyte differentiation in vivo, which might contribute to the thrombocytopenia of leukemia mice [108]. Our preclinical data using pharmacological approaches to inhibit IL-4 in combination with AraC treatment showed that targeting IL-4 represents a promising strategy to improve the therapeutic responses in leukemia [108]. Additionally, anti-IL-4 has been shown to be safe when administered to patients with asthma [109], implying that it could be applied in the treatment of thrombocytopenia. However, all the data were generated based on a specific leukemia model and it is unclear whether results obtained can be generalized to CIT. More studies are, therefore, needed to expand this paradigm to CIT and to explore whether our findings in the mouse model can be translated to human. The findings of the current study suggest that CIT in cancer patients results from HSCs losing their capacity for self-renewal, HPCs apoptosis, and BMECs dysfunction (Fig. 1). For terminal megakaryocyte maturation and normal platelet production, by using a culture system that recapitulates in vitro human megakaryopoiesis, Ann Zeuner et al. [110] found that cytotoxic drugs mainly destroyed megakaryocytic progenitors at early stages of differentiation by inducing apoptosis, and cytokine stem cell factor (SCF) can protect immature megakaryocytes from the influence of chemotherapy drugs. Future research must examine how tumor cells affect BM niche and educate HSCs.

## Data Availability

Not applicable.
